# Impact on hospitals of price reductions for physician-administered biologics

**DOI:** 10.1093/haschl/qxag090

**Published:** 2026-04-16

**Authors:** James C Robinson, Mark Thomson, Ari D Kosorukov, Christopher M Whaley

**Affiliations:** University of California, School of Public Health, Berkeley, Berkeley, CA 94720-7360, USA; University of California, School of Public Health, Berkeley, Berkeley, CA 94720-7360, USA; University of California, School of Public Health, Berkeley, Berkeley, CA 94720-7360, USA; Brown University, School of Public Health, Providence, RI 02912, USA

**Keywords:** physician-administered drugs, biologics, price reductions, hospital margins, private insurers, buy-and-bill, Inflation Reduction Act, Most Favored Nation, biosimilar competition

## Abstract

The prices of physician-administered drugs and biologics are coming under downward pressures from Medicare price negotiations, Most Favored Nation policies, and competition from biosimilars. These will reduce manufacturer sales revenue but potentially increase revenues for the hospitals that acquire these products at one price and are reimbursed by insurers at a higher price.

This paper uses 2020-2024 Blue Cross Blue Shield insurer data on expenditures, pricing, and utilization to estimate the impact of manufacturer price decreases for 20 major biologics.

Hospital margins for these 20 products increased from $2.37 billion in 2020 (55% of insurer expenditures) to $2.97 billion in 2024 (59%). A 20% manufacturer price reduction would increase hospital buy-and-bill margins to $4.84 billion, 52% of insurer expenditures, while a 40% reduction would increase hospital margins to $5.97 billion, 64% of insurer expenditures. The shift in insurer expenditures from drug manufacturers to hospitals is estimated to reduce research and development (R&D) investments between $282 and $564 million in 2028.

A large share of insurer expenditures for physician-administered drugs and biologics are retained by hospital intermediaries rather than accruing to pharmaceutical manufacturers.

The prices of physician-administered drugs and biologics are coming under pressure from the Inflation Reduction Act (IRA), Most Favored Nation (MFN) policies, and competition from therapeutically equivalent generics and biosimilars. Downward pressures on prices will reduce revenues for pharmaceutical manufacturers but potentially increase revenues for the hospitals that acquire these products at one price and are reimbursed by insurers at a higher price, in a process often referred to as “buy-and-bill.” Although Medicare reduces its payments to hospitals commensurate with reductions in drug acquisition prices, private insurers negotiate payment rates without direct regard to the amounts paid by the hospitals to manufacturers. Reductions in acquisition prices without offsetting reductions in reimbursement increase hospital revenues in a manner analogous to the hospital margin expansions obtained through the federal 340(B) drug price discount program.

This paper analyzes the potential impact on hospitals of price reductions for physician-administered drugs, with a focus on private insurers. It begins with a description of price pressures from the IRA, MFN policies, and biosimilar competition, and then describes the differential impacts for patients covered by Medicare and those covered by private insurance. The paper uses data on private insurer expenditures, infusion volumes, reimbursement prices paid by insurers to hospitals, and acquisition prices paid by hospitals to manufacturers for 20 biologics that collectively account for the largest portion of Medicare spending on physician-administered pharmaceuticals. It calculates revenue gains by hospitals and losses for manufacturers between 2020 and 2024 and projects the potential increase in hospital buy-and-bill revenues in 2028. It estimates the potential reduction in manufacturer R&D investments due to the diversion of insurer expenditures from manufacturers to hospitals.

## Pricing and reimbursement for biologics

### Downward pressures on prices

Downward pressures on the prices for physician-administered biologics are coming from Medicare price negotiations, the development of MFN policies, and the market share gains by less expensive biosimilars. These pressures will affect individual biologics in different ways, depending on inclusion and exclusion criteria for the IRA negotiations, the differences between United States and foreign prices for MFN policies, and the interest among biosimilar manufacturers in entering each product market. The 3 different pricing pressures are complementary in the sense that those biologics most likely to be affected by one class of pressures will be least likely to be affected by the others, and vice versa. For example, while some biologics will be unlikely to face market entry and competition from biosimilars, they will face pressures that are not contingent on biosimilar entry. Biologics that face biosimilar competition are explicitly exempted from Medicare price negotiations and MFN pricing principles will be applied most rigorously to newly launched biologics, precisely the ones least likely to face biosimilars.

In 2026, Medicare implemented price reductions for patient-administered drugs covered under Part D, negotiated under provisions of the Inflation Reduction Act, and will implement a new set of reductions in 2027. Beginning in 2028, it will extend price reductions to physician-administered biologics covered under Part B.^1,2^ For drugs covered under Part D, Medicare negotiates with manufacturers the prices that Medicare itself will pay the manufacturers. For biologics covered under Part B, however, Medicare will negotiate with manufacturers the prices that the manufacturers will be paid by hospitals, which only subsequently will be reimbursed by Medicare.

The manner with which MFN principles will be applied to physician-administered drugs and biologics remains to be determined. The Centers for Medicare and Medicaid Innovation (CMMI) has announced a demonstration project to apply MFN principles to Part B, but design details have yet to be provided.^3^ The first Trump administration proposed an analogous policy in the form of the “international pricing initiative” (IPI).^4,5^ This would have capped Medicare Part B prices at 25% above the average paid in other developed nations. The IPI would have avoided the complications of buy-and-bill reimbursement by requiring drug manufacturers to sell their products to a new class of organizational intermediaries, at IPI prices. The intermediaries would distribute the drugs as needed, without the providers taking ownership. The IPI initiative was not implemented, for multiple reasons, but politically complicating factors included the administrative difficulty of contracting with new intermediaries and the hospitals' loss of buy-and-bill revenues. For purposes of this analysis, we assume that MFN policy will retain the buy-and-bill structure of distribution and reimbursement.

The third downward pressure on the prices of physician-administered biologics stems from market share gains by biosimilars. The adoption of biosimilars for patient-administered drugs has lagged due to the preference of the organizational intermediaries in the retail drug channel, Pharmacy Benefit Managers (PBM), for products that charge high prices (to the insurers) but offer large rebates (to the PBMs).^6^ In the institutional drug channel, however, hospital intermediaries have embraced biosimilars as they have been able to negotiate high reimbursement price markups. The share gains by biosimilars have been accompanied by fierce competition and rapid declines in manufacturer prices.^7,8^

### Acquisition prices and reimbursement payments

Physician-administered pharmaceuticals include biologics, gene and cell therapies, chemotherapies, and other complex molecules that cannot be taken by pill, capsule, or self-injection. Biologics are by far the largest component of the class and include the largest 20 products in terms of annual expenditures. These products are not distributed through retail and mail order pharmacies but, rather, through hospitals and physician practices. Providers purchase products from manufacturers at one price and then are reimbursed by insurers at a second, higher price. These buy-and-bill margins come at the expense of drug manufacturers, who receive less revenue from insurers than they would if a portion were not diverted to the intermediaries.

For patients covered by Medicare, downward pressure on manufacturers' prices will reduce provider margins and generate savings for the public program. Under Part B, Medicare reimburses providers an amount equal to the average sales price (ASP) obtained by manufacturers from all purchasers and adds a 6% markup to cover the providers' costs of handling and inventory. When IRA negotiations, MFN policies, and biosimilar competition force manufacturers to reduce acquisition prices, Medicare will reduce its reimbursements commensurately. Medicare will reap savings from this dynamic, since it will pay less for the drugs themselves and less for the 6% markup.

The impact of drug price reductions on hospitals and physician practices will be quite different for patients covered by private insurers, since there is no automatic linkage between hospital procurement spending and subsequent reimbursement from insurers. Providers will pay lower acquisition prices for the drugs they administer to patients covered by private insurance, as well as for those covered by Medicare, since drugs are acquired without respect to the patients who eventually access them. Given their strong bargaining position with insurers, hospitals have been able to negotiate reimbursement prices 250%-350% above the acquisition prices they pay to manufacturers.^9^ There is no requirement for hospitals to pass to insurers the value of any decline in acquisition prices. Insurers may negotiate reductions in reimbursement prices over time, but likely at a rate slower than the decline in acquisition prices, allowing for a growth in hospital markups and margins.

A growing spread between reimbursement prices and acquisition prices has been observed in the context of market entry and competition between physician-administered biosimilars and biologics.^10^ An analogous growing spread between list and net prices has been reported for patient self-administered products, including insulins, which are distributed through retail pharmacies rather than the buy-and-bill distribution channel but which also strongly influenced by supply chain intermediaries. An analysis of 2015 industry data reported by 41% of expenditures for pharmaceuticals accrued to supply chain intermediaries, including wholesalers, pharmacies, pharmacy benefit managers, and insurers, with the remainder accruing to manufacturers.^11^ Between 2014 and 2018 the list prices charged by insulin manufacturers to insurers grew by 40% but the net prices received by the manufacturers, after accounting for revenue retention by Pharmacy Benefit Managers, wholesale distributors, and retail pharmacies, decreased by 31%. The share of total insurer expenditures for these products declined from 70% to 47% during this period.^12^

Further insights into the potential impact on hospitals of price reductions by manufacturers can be obtained from the experience of the federal 340(B) drug discount program. Under terms of the 340(B) program, drug manufacturers are required to extend to eligible hospitals the discounts they are otherwise mandated to offer state Medicaid programs. These low prices are paid for drugs administered to privately insured and Medicare patients as well as to the uninsured and patients covered by Medicaid. Centers for Medicare and Medicaid Services (CMS) has estimated the value of the 340(B) discounts as 35% of the manufacturer's ASP.^13^ Eligible hospitals thus purchase drugs at prices substantially below ASP but are reimbursed by Medicare at rates above ASP. In 2021, Medicare sought to claw back these margins by reducing the reimbursement it pays to eligible hospitals but was forced by the courts to reinstate the higher rate.^14,15^ A similar effect likely will occur when IRA negotiations, MFN policies, and biosimilar competition reduce the acquisition prices paid by hospitals without automatically reducing the reimbursement prices paid to them by private insurers.

The retention by hospitals and other distribution chain intermediaries of insurer expenditures reduces the sales revenues accruing to drug manufacturers and, in turn, industry investments in research and development. In 2021 the global pharmaceutical industry, across all regions and firm sizes, invested 27% of sales revenues in R&D. US drug firms invested 34% of sales revenues.^16^ These figures exceed a previously published 21% estimate because they include investments by small and midsized firms, which exhibit a higher research investment intensity than larger corporate entities. The biologics included in this study are manufactured by large firms and we use 25% as our estimate of the percentage of revenues that in invested. We use this as the basis for our estimates of reductions in investment following reductions in revenues.

## Data

This study is based on 2020-2024 claims data for the 20 biologics that collectively account for over half of Medicare Part B expenditures and were profiled in a report by the US Assistant Secretary for Planning and Evaluation (ASPE).^17^ Criteria for inclusion in IRA price negotiations include scale of Medicare expenditures and orphan disease designation, among others, and the selection was announced by CMS early in 2026.^18^ Inclusion criteria for the MFN demonstration project are under development but likely will focus on high-expenditure products and those newly launched onto the market. Exposure to market entry and competition from biosimilars will depend on patent extension strategies pursued by manufacturers of biologics and on the size of the potential market, among other factors. It is impossible to predict which biologic will be affected most by which type of price pressure, and for purposes of this study we assume they are equally exposed.

De-identified insurance claims were obtained for privately insured patients enrolled in Blue Cross Blue Shield health plans nationally, obtained through the database maintained by the Blue Cross Blue Shield Association.^19^ The data cover patients covered by commercial insurance, including public and private employers, self-insured firms, labor unions, and individuals enrolled through the state and federal health insurance exchanges. The study does not include individuals enrolled in Medicare or Medicaid. The claims include the code for the biologic, number of units infused, amount reimbursed by the insurer, patient principal diagnosis (ICD-10 code), and provider identifiers. We limited the analysis to biologics administered through hospital outpatient departments to the exclusion of those administered in community-based physician practices. We also exclude biologics administered to hospital inpatients.

The reimbursement price was measured in terms of paid amount divided by the number of units administered. This is the actual price paid by the insurer to the hospital, not the manufacturer's nominal “wholesale acquisition price” or the hospital's nominal “chargemaster” price. The acquisition price paid by hospitals for each biologic was estimated using the ASP files published by CMS on a quarterly basis.^20^ ASP represents the average price received by manufacturers from all purchasers, after accounting for discounts and rebates, not the amount paid by each individual hospital. We did not have data on the acquisition price paid by individual facilities.

## Methods

For each year we calculated the number of patients receiving infusions, the number of infusion visits, and the number of units infused, for each of the 20 biologics. Insurer expenditures were calculated by summing the paid amounts from the claims for each of the biologics and for the 20 as a group. Manufacturer revenues were calculated by multiplying the number of units sold by the average acquisition price for each biologic. Hospital margin revenues were calculated by subtracting manufacturer revenues from insurer expenditures.

IRA negotiations obtained price discounts of 22% off the net prices paid for 10 patient-administered drugs in the first year and 44% discounts off net prices for 15 drugs in the second year.^21,22^ A 40% price reduction likely would result if MFN principles are applied to US drug prices, given that foreign prices have been found in government-sponsored studies to exceed US prices by 250%.^23^ Industry analyses report that the market prices of biologics and biosimilars declined by up to 50% in their first years facing biosimilar competition.^24,25^ For purposes of this study, we estimate the impacts of 20% and 40% price reductions, respectively, in net manufacturer prices for physician-administered biologics between 2024 and 2028.

We project 2028 manufacturer revenues under the assumption that IRA negotiations, MFN policies, and biosimilar competition reduce manufacturer prices but that hospitals do not acquiesce to similar reductions in the reimbursement prices they receive from private insurers. We do not adjust these estimates for the effect, if any, of price declines on the volume of biologics administered. Provisions of the IRA are expected to increase sales volumes by reducing beneficiary cost sharing obligations. Reductions in consumer cost sharing due to lower MFN prices and price competition from biosimilars may increase utilization.

## Results

Table 1 presents 2020-2024 trends in patient utilization, insurer expenditures, hospital buy-and-bill revenues, and hospital revenues as a percent of insurer expenditures for the 20 biologics as a group. The number of patients and infusion visits remained relatively stable over the 5 years, but drug volumes, measured in terms of units infused, increased by 20%. Blue Cross Blue Shield insurers increased their annual expenditures on these biologics by 16%, from $4.31 billion in 2020 to $5.02 billion in 2024. This increase in insurer expenditures was accompanied by an increase in the hospital margin revenues from $2.37 billion (55% of insurer expenditures) in 2020 to $2.97 billion (59%) in 2024. Hospital revenues from theses 20 biologics across the 5-year period summed to $13.60 billion.

**Table 1.** Trends in utilization, insurer expenditures, and provider revenues for 20 physician-administered biologics.

	2020	2021	2022	2023	2024	2020-2024 total
**Patients treated**	62 892	61 228	57 920	57 348	57 715	202 240
**Infusion visits**	266 093	262 665	267 082	267 228	269 485	1 332 553
**Units infused (1000)**	48 079	51 804	58 330	59 139	57 732	275 084
**Insurer expenditures** **($ millions)**	4310	4407	4868	4977	5019	23 581
**Provider revenues** **($ millions)**	2372	2480	2841	2933	2971	13 597
**Provider revenues as percent of insurer expenditures**	55	56	58	59	59	58

Source: Claims data from Blue Cross and Blue Shield plans nationally.

Appendix Table S1 presents the number of patients treated, the principal disease indications addressed, the number of drug infusion visits, number of drug units infused, and insurer expenditures for each of the 20 biologics and for the 20 products combined. During the 5 years covered by this study, 202 240 Blue Cross and Blue Shield enrollees incurred 1.33 million infusion visits for these 20 biologics, for which the insurers paid $23.58 billion. Expenditures were concentrated in a subset of products, including Keytruda ($6.80 billion), Ocrevus ($3.98 billion), Opdivo ($2.78 billion), Darzalex IV and SubQ ($1.85 billion), Entyvio ($1.40 billion), Remicade ($1.27 billion), and Rituxan ($1.04 billion).

**Figure 1.**
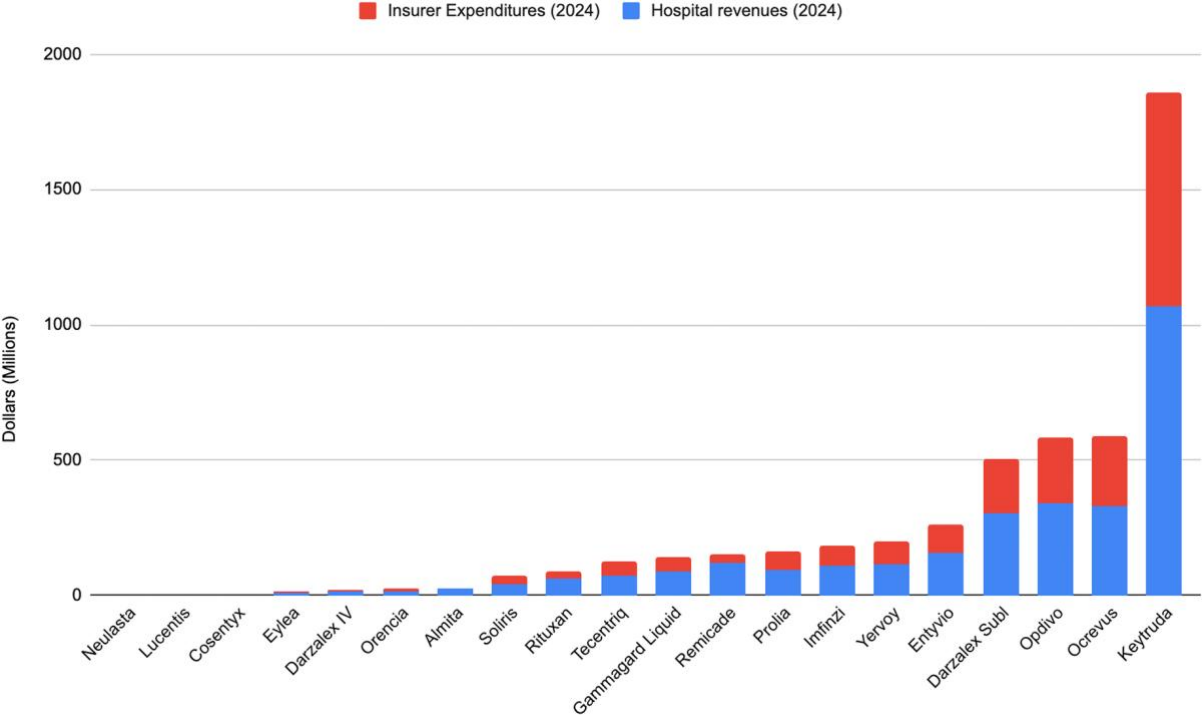
Insurer spending and hospital revenue for 20 major biologic drugs. Source: claims data from Blue Cross and Blue Shield plans nationally. Bar height reflects 2024 values: one set shows hospital revenue from price markups on these biologics, while the other shows total insurer spending, inclusive of those markups.

Figure 1 presents 2024 insurer expenditures and hospital margin revenues for each of the 20 biologics. Hospital revenues range widely, from lows of $1000 for Neulasta and $380 000 for Lucentis to highs of $329 million for Ocrevus and $1.07 billion for Keytruda. The share of insurer expenditures retained by hospitals as buy-and-bill margins also ranges widely, from lows of 10% for Neulasta to highs of 90% for Almita and 80% for Remicade.

Appendix Table S2 presents estimated hospital margins for 2028 under the assumption that IRA negotiations, MFN policies, and biosimilar competition reduce prices by 20% and 40%, respectively, from 2024 levels. A 20% manufacturer price reduction would increase hospital buy-and-bill margins from $3.71 billion to $4.84 billion, 30% above 2024 levels. If market and regulatory pressures reduced prices by 40%, hospital revenues would increase to $5.97 billion, 61% above 2024 levels.

**Figure 2.**
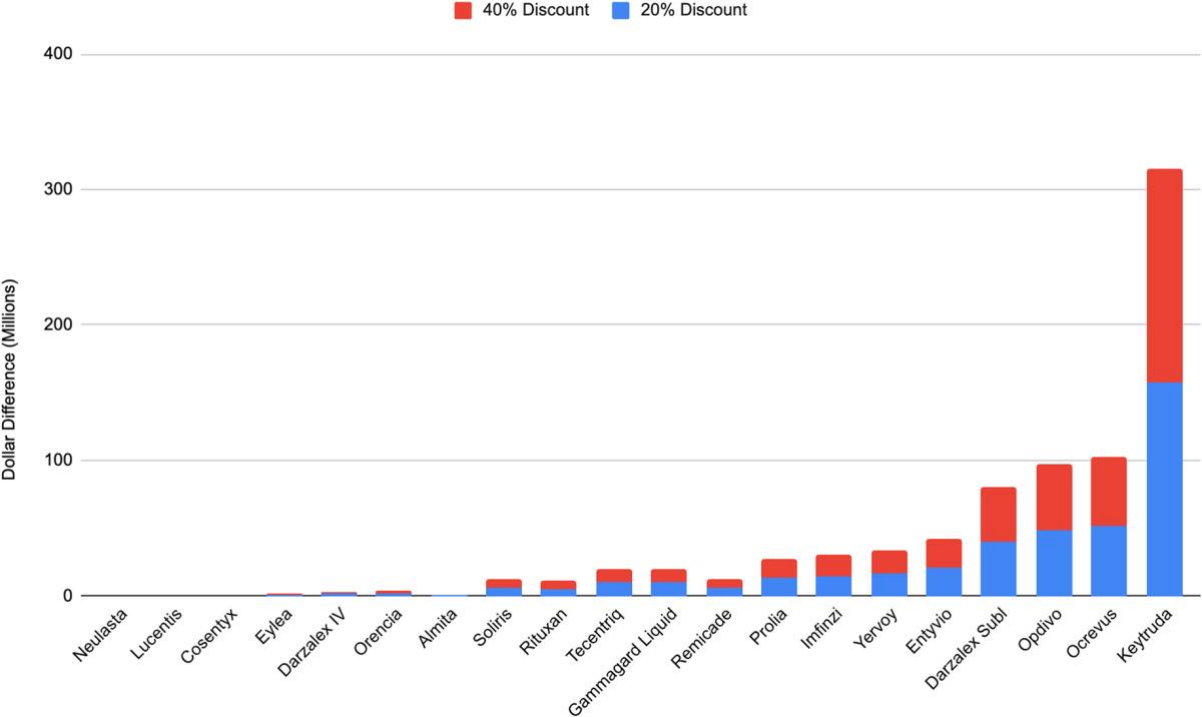
Increases in hospital revenues related to 20% and 40% manufacturer price reductions for 20 major biologics. Source: Claims data from Blue Cross and Blue Shield plans nationally. Bar height reflects the estimated revenue gains in 2028 relative to 2024: one set shows the increase associated with a 20% price reduction, while the other shows the increase associated with a 40% reduction, inclusive of the smaller increase.

The projected increases in hospital revenues resulting from manufacturer price reductions for each of the 20 biologics are illustrated in Figure 2. These also vary extensively. At a 20% manufacturer price reduction, hospital margin revenues from Keytruda would increase by $291 million, from $1.187 billion to $1.478 billion. At a 40% price reduction, hospital revenues would increase by $583 million, to $1.770 billion. A 20% manufacturer price reduction for Ocrevus would increase hospital revenues by $201 million, from $470 million to $671 million, while a 40% price reduction would increase hospital revenues by $402 million to $872 million. Conversely, manufacturer price reductions for Almita, Neulasta, Lucentis, and other drugs with low insurer expenditures would not substantially affect hospital margin revenues.

Increases in hospital revenues would come at the expense of drug manufacturers and reduce their capability to invest in R&D. We assume that the manufacturers of these biologics would invest in R&D at the same rate as the industry as a whole, 25% of sales revenues. If prices in 2028 are 20% lower than in 2024, with a resulting $1.13 billion increase in hospital buy-and-bill margin revenues, manufacturers are projected to reduce R&D investment by $282 million (25% of the $1.13 billion) compared to what would have been invested had prices remained at 2024 levels. If 2028 prices are 40% below 2024 levels, manufacturers are projected to reduce R&D investment by $564 million, 25% of the $2.26 billion increase in hospital margin revenues.

## Limitations

The results of this study should be interpreted considering its limitations. The data derived from Blue Cross and Blue Shield health plans, which may not be representative of other private insurers, and do not include patients covered by Medicaid and Medicare. We focus on the largest physician-administered products, in terms of expenditures, but must make assumptions when projecting from the 2020 to 2024 period, for which we have data, to the 2028 year, for which we do not. IRA negotiations, MFN policies, and biosimilar competition will affect the 20 biologics in ways difficult to predict, given likely changes in legislative, regulatory, and market factors. Our assumptions of 20% and 40% reductions in manufacturer prices may overstate or understate the impact on individual products. We cannot predict changes in infusion volumes that may result from the projected changes in price and hence affect our estimates of changes in manufacturer revenues.

## Discussion

As reported in this paper, hospitals retained 58% of Blue Cross and Blue Shield insurer expenditures for 20 major biologics over the 2020-2024 period. The diversion of insurer spending from manufacturers to hospitals likely will increase as the IRA negotiations are extended, MFN policies are developed, and biosimilars gain market share in the years leading up to 2028. The downward pressures on biologic prices are complementary. For example, while competition from biosimilars will affect some biologics extensively and other biologics only to a limited extent. the other pressures are not contingent on biosimilar entry and, indeed, will be strongest for products where biosimilar competition is weakest. Under terms of the IRA, biologics that face biosimilar competition are explicitly exempted from Medicare price negotiations. Most Favored Nation pricing principles will be applied most rigorously to the prices of newly launched biologics, the ones least likely to face competition from biosimilars.

This study did not attempt to quantify the impact, if any, of the hospital markups on the out-of-pocket cost sharing required of patients using these products. The structure of cost sharing for biologics covered under the commercial medical benefit is complex, frequently involving annual deductibles and percentage coinsurance as well as dollar copayments. The intensity of the requirements varies considerably among self-insured employers and labor unions as well as among insurers for their fully insured clients. Deductible-based benefit designs create a strong inter-temporal variation in cost sharing, with out-of-pocket payments declining over the course of the year as more patients meet their deductible obligations. A recent study found no consistent association between the advent of biosimilar competition, on the one hand, and the extent of consumer cost sharing, on the other.^26^

It is useful to compare the experience of the Blue Cross and Blue Shield insurers to that of the Medicare program. As reported by ASPE for 2021, Medicare spent $16.3 billion on these 20 biologics, treating 1.49 million beneficiaries.^27^ Providers were entitled to retain 6% of these expenditures ($978 million), while the remainder, $16.2 billion, accrued to the drug manufacturers. If the manufacturers invested at the industry average (25%), the 2021 foregone Medicare sales revenues would have financed $245 million in R&D.

This pales with respect to the investment foregone due to hospital revenues from the private insurers included in this study. In 2021, the Blue Cross and Blue Shield plans spent $4.41 billion on these 20 biologics for the treatment of 206 355 patients. Under the assumption that drug manufacturers invest 25% of revenues, the $2.48 billion retained by hospitals and not earned by manufacturers in that year would have financed $620 million in additional R&D.

Substantial impacts on investment would be expected if IRA negotiations, MFN policies, and biosimilar competition reduce 2028 manufacturer prices from 2024 levels. In that case, hospital drug margins from private insurers would be even higher, and foregone manufacturer R&D investment would be even greater. It should be emphasized that our 25% estimate of manufacturer research intensity is an industry average and not specific to the firms producing the biologics included in this study. The relationship between current revenues, projected future revenues, and R&D investment is complex and varies among firms and across time periods. Our estimates thus should be interpreted accordingly. Nevertheless, any substantial diversion of manufacturer sales revenues to hospital margins cannot but impact manufacturer investments and, over time, product innovation.

As documented in this study, a large share of insurer expenditures for physician-administered drugs and biologics are retained by hospital intermediaries rather than accruing to pharmaceutical manufacturers. Hospitals need supplementary revenues, beyond those obtained for their clinical services, to cover the costs of treating all patients regardless of insurance coverage and ability to pay. However, reliance on drug price markups and margins motivates hospitals to expand drug distribution and administration through the acquisition of physician practices, rather than their core clinical services, a questionable incentive from the perspective of the health care system as a whole.

## Supplementary Material

qxag090_Supplementary_Data
