# Supplementary material for: Impact on hospitals of price reductions for physician-administered biologics
Source: Health Aff Sch. 2026 Apr 16;4(4):qxag090. doi: 10.1093/haschl/qxag090 (PMC13126657; doi:10.1093/haschl/qxag090)
Supplement: qxag090_Supplementary_Data [file qxag090_supplementary_data.zip › Appendix Table 1.docx]

**Appendix Table 1**

**Expenditures, Utilization, Prices, and Hospital Revenues for 20 Major Biologics, 2020-24**

| **Biologic (name)** | **Unique Patients Treated** | **Principal Patient Indications** | **Number of Infusion Visits** | **Number of Units Infused** | **Insurer Expenditures (Millions)** |
| --- | --- | --- | --- | --- | --- |
| **All 20 biologics (Sum of 20 rows)** | 202,240 |  | 1,332,553 | 275,084,276 | 23581.47 |
| **Almita** | 9,306 | Cancer | 51,359 | 5,529,830 | 555.51 |
| **Cosentyx** | 259 | Inflammatory  disease | 21,06 | 853,756 | 11.15 |
| **Darzalex IV** | 1,821 | Cancer | 20,077 | 3,264,318 | 356.97 |
| **Darzalex Subl** | 6,081 | Cancer | 79,207 | 16,665,472 | 1495.00 |
| **Entyvio** | 16,003 | Inflammatory Disease | 109,148 | 37,366,884 | 1395.07 |
| **Eylea** | 3,883 | Eye disease | 206,19 | 57,606 | 79.97 |
| **Gammagard Liquid** | 7,516 | Immune  system  deficiency | 63,721 | 6,405,494 | 570.73 |
| **Imfinzi** | 4,956 | Cancer | 36,156 | 4,885,978 | 700.08 |
| **Keytruda** | 36,836 | Cancer | 276,035 | 70,388,987 | 6796.52 |
| **Lucentis** | 504 | Eye Disease | 2,313 | 12,404 | 6.85 |
| **Neulasta** | 7,830 | Cancer | 17,846 | 158,352 | 335.67 |
| **Ocrevus** | 19,297 | Inflammatory Disease | 52,580 | 30,592,684 | 2976.19 |
| **Opdivo** | 16,156 | Cancer | 127,332 | 50,790,935 | 2777.17 |
| **Orencia** | 1,998 | Inflammatory Disease | 16,988 | 1,529,038 | 128.25 |
| **Prolia** | 45,159 | Osteoporosis | 189,794 | 21,289,687 | 858.43 |
| **Remicade** | 19,654 | Inflammatory Disease | 142,069 | 9,651,384 | 1268.13 |
| **Rituxan** | 16,603 | Inflammatory Disease | 59,736 | 5,792,362 | 1043.33 |
| **Soliris** | 736 | Inflammatory  disease | 10,068 | 1,336,225 | 481.70 |
| **Tecentriq** | 5,133 | Cancer | 37,109 | 5,095,314 | 764.60 |
| **Yervoy** | 6,482 | Cancer | 18,380 | 3,417,567 | 980.15 |
