# Supplementary material for: Impact on hospitals of price reductions for physician-administered biologics
Source: Health Aff Sch. 2026 Apr 16;4(4):qxag090. doi: 10.1093/haschl/qxag090 (PMC13126657; doi:10.1093/haschl/qxag090)
Supplement: qxag090_Supplementary_Data [file qxag090_supplementary_data.zip › Appendix Table 2.docx]

**Appendix Table 2**

**Expenditures, Utilization, Prices, and Hospital Revenues for 20 Major Biologics**

| **Biologic (name)** | **Insurer expenditures (2024)** | **Units infused (2024)** | **ASP (average for 2024)** | **Hospital revenues (2024)** | **Hospital revenue at 20% price discount** | **Hospital revenue at 40% price discount** |
| --- | --- | --- | --- | --- | --- | --- |
| **All 20 biologics (Sum of 20 rows)** | 5,018.50 | 57,732,537 |  | 2,970.61 | 3,380 | 3,790 |
| **Almita** | 27.76 | 882,541 | 4.55 | 24.71 | 25.32 | 25.93 |
| **Cosentyx** | 2.05 | 162,640 | 4.64 | 1.43 | 1.55 | 1.67 |
| **Darzalex IV** | 20.77 | 172,196 | 62.77 | 12.72 | 14.33 | 15.94 |
| **Darzalex Subl** | 505.73 | 5,282,791 | 50.15 | 306.00 | 345.95 | 385.89 |
| **Entyvio** | 263.88 | 6,662,598 | 22.26 | 156.97 | 178.35 | 199.73 |
| **Eylea** | 13.59 | 9,614 | 840.62 | 7.59 | 8.79 | 9.99 |
| **Gammagard Liquid** | 141.53 | 1,536,609 | 45.54 | 90.96 | 101.07 | 111.19 |
| **Imfinzi** | 184.20 | 1,256,748 | 81.27 | 107.57 | 122.89 | 138.22 |
| **Keytruda** | 1,857.27 | 18,355,780 | 57.01 | 1,069.16 | 1,226.78 | 1,384.40 |
| **Lucentis** | 0.50 | 1,018 | 159.80 | 0.38 | 0.40 | 0.43 |
| **Neulasta** | 0.01 | 108 | 83.50 | 0.00 | 0.00 | 0.00 |
| **Ocrevus** | 586.95 | 5,889,294 | 59.62 | 329.10 | 380.67 | 432.24 |
| **Opdivo** | 584.89 | 10,235,538 | 31.39 | 341.54 | 390.21 | 438.88 |
| **Orencia** | 26.70 | 317,915 | 43.29 | 16.56 | 18.59 | 20.61 |
| **Prolia** | 162.59 | 3,510,339 | 26.04 | 93.14 | 107.03 | 120.92 |
| **Remicade** | 153.03 | 1,312,748 | 31.94 | 122.22 | 128.38 | 134.54 |
| **Rituxan** | 88.74 | 500,845 | 78.28 | 60.11 | 65.83 | 71.56 |
| **Soliris** | 73.20 | 200,672 | 225.39 | 40.27 | 46.86 | 53.44 |
| **Tecentriq** | 123.93 | 803,658 | 85.22 | 72.52 | 82.80 | 93.08 |
| **Yervoy** | 201.18 | 638,885 | 174.80 | 117.67 | 134.37 | 151.07 |
